# 25-Hydroxyvitamin D Supplementation and BMI Change: A Meta-Analysis of Randomized Controlled Trials

**DOI:** 10.4172/2165-7904.1000181

**Published:** 2013-07-09

**Authors:** N Mora, K Rieke, J Plitcha, AJ Segura, D Leehey, K DeShong, H Kramer, Ramon A. Durazo-Arvizu

**Affiliations:** 1 Public Health Program, Loyola University Chicago Health Sciences Center, Chicago, Illinois, USA; 2 Departments of Surgery, Loyola University Chicago Health Sciences Center, Chicago, Illinois, USA; 3 Medicine, Division of Nephrology and Hypertension, Loyola University Chicago Health Sciences Center, Chicago, Illinois, USA; 4 Department of Preventive Medicine and Epidemiology, Loyola University Chicago Health Sciences Center, Chicago, Illinois, USA

**Keywords:** 25-hydroxyvitamin D (25[OH]D), Vitamin D, Body Mass Index (BMI), Obesity, Meta-analysis

## Abstract

The impact of 25-hydroxyvitamin D (25[OH]D) supplementation on weight change remains controversial. The objective of this study was to summarize the effects of 25[OH]D supplementation (cholecalciferol or ergocalciferol) on BMI change through a meta-analysis of published clinical trials. We completed a systematic review of English articles, using MEDLINE (Ovid, Pubmed) from January 1, 1998 through January 1, 2013. The articles selected focused on 25[OH]D supplementation and body mass index (BMI) in randomized controlled trials (RCT’s). The association between 25[OH]D and mean BMI change was estimated utilizing a random effects model. A total of 30 studies were reviewed and 9 were included in the meta-analysis. Total participants included 1651 adults (82.6% women and mean age 47.9 years) and mean follow-up ranged between 6 to 196 weeks and mean daily 25[OH]D dose ranged from 200 IU to 1,110 IU. Five of the 9 studies included calcium supplementation in both groups. Average baseline BMI was 30.7 and 30.4 kg/m^2^ in the intervention and control groups, respectively. Five studies suggested a beneficial effect for 25[OH]D supplementation for BMI change whereas 3 studies showed no effect of 25[OH]D supplementation on BMI change, and one showed a non-perceptible change. Meta-analysis of BMI values at end of trial showed no statistically significant difference in BMI change by use of 25[OH]D supplementation. Based on existing published trials, oral 25[OH]D supplementation does not significantly impact BMI change.

## Introduction

Low calcium intake and insufficient or deficient 25-hydroxyvitamin D (25[OH]D) levels are often observed in obese patients [1]. Consequently, interest in potential mechanisms by which calcium and 25[OH] D regulate body weight has increased. Evidence suggests that calcium and 25[OH]D increase whole body fat oxidation after single and multiple meals, and that calcium promotes a modest energy loss through increased fecal fat excretion [2]. The evidence is equivocal for a greater diet-induced thermogenesis, increased lipolysis, suppression of key lipogenic enzymes, decreased hunger or reduced energy/macronutrient intake [3]. The active form of vitamin D, 1,25-dihydroxy-vitamin D3, appears to decrease adipogenesis [4]. However, experiments in vitamin D receptor (VDR) null mice suggest a role for 25[OH]D to impair energy expenditure [5]. Overall, the role of 25[OH]D on energy expenditure and fat oxidation in humans remains controversial [6]. Emerging evidence suggests a potential improvement in insulin sensitivity following 25[OH]D supplementation that could increase substrate oxidation and promote weight loss. However, whether low levels of 25[OH]D are a cause or effect of insulin resistance and obesity remains unclear [7]. In addition, suppression of parathyroid hormone (PTH) by 25[OH]D supplementation could have a beneficial effect in obesity [8].

Several randomized controlled trials (RCTs) have evaluated the effect of 25[OH]D on weight loss. In a recent systematic review, the authors considered the data to be inadequate to draw conclusions and a meta-analysis was not attempted [9]. However, this review did not include data from studies published after 2010. Therefore, we performed a systematic review and meta-analysis of all RCTs published from January 1, 1998 to January 1, 2013 which compared 25[OH]D supplementation (cholecalciferol, or ergocalciferol) to placebo and had BMI change as either a primary or secondary outcome.

## Methods

### Literature search strategy

We conducted a language restricted systematic review of all articles published in English using MEDLINE (Ovid, Pubmed) from January 1, 1998 through January 1, 2013 on 25[OH]D and BMI in randomized controlled trials. For identifying interventions containing our main analysis, the following keywords or MeSH terms were used: randomized controlled trials, random effects models, double-blinded method, cholecalciferol, ergocalciferol, dihydroxycholecalciferols or calcifediol, vitamin D supplementation or intervention, body mass index, weight loss, weight change, overweight, obesity (Appendix A). Any relevant references cited in the selected articles, abstracts, or books were selected via manual research. Any articles containing keywords were reviewed. From these articles, those that reported randomized controlled trials were considered and examined by three of the authors (A.J.S., N.M., and J.P.).

### Eligible studies

**Figure 1:**
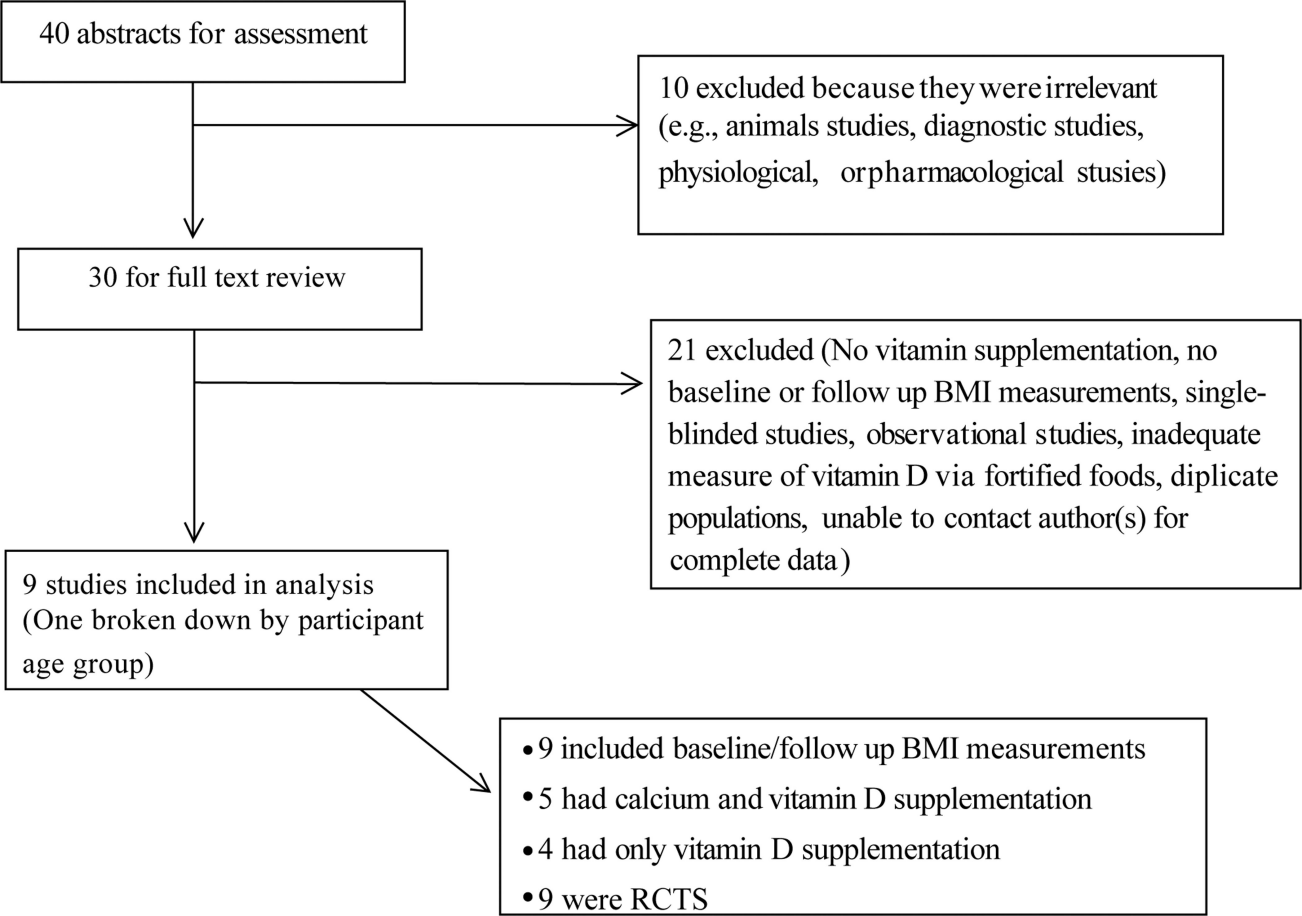
Flow chart of studies selected.

We included only RCTs that studied orally administered 25[OH] D supplementation (cholecalciferol, or ergocalciferol) versus a placebo. Use of calcium supplements as the primary intervention strategy with 25[OH]D as a secondary supplement or vice versa was not restricted in our primary analysis. To be included in the primary analysis, we required reported administration of 25[OH]D supplementation and dosage, and baseline and follow-up BMI measurements. There was no restriction on age, gender, geographic location, or time of follow for study participants. We excluded uncontrolled trials, observational studies, and animal studies. Any studies that did not provide adequate or precise 25[OH]D administration (such as through 25[OH]D fortified foods or dietary sources of 25[OH]D) were not included. Those that did not contain a measureable or obtainable baseline or follow up BMI along with respective standard errors were excluded. Figure 1 shows the study selection process based on these criteria.

### Data extraction

Data were extracted independently by three reviewers (A.J.S., N.M., and J.P.). Guidelines for abstracting data were established for optimal data extraction and a data spreadsheet template was generated for ease of use and commonality between reviewers. Any disagreements or contradictions in obtained data were resolved by re-review of literature by reviewers to come to a consensus. Information extracted included study year, author(s), study location, study design, primary and secondary outcomes, trial duration, mean follow up (in weeks), 25[OH] D and calcium doses (for intervention and placebo groups), number of study participants, mean age, percent female/male, and baseline and follow up BMI. Authors were contacted if additional study details or data were needed.

### Quality assessment

The quality of each study was assessed using the scale provided in Martyn and Carroll [10] and a modified version of the scale provided in Downs and Black [11]. The Jadad scale consisted of three questions with a total score ranging from 0–5. The modified Downs scale consisted of 27 questions with total scores ranging from 0–28. For the Downs scale, question 27 was modified to a 0 or 1 scoring system based on whether or not a power calculation was reported. Three reviewers (K.D., D.L., and K.R.) independently rated the quality of all studies using both scales. In the case of discrepancies in item scores, the majority rated score was used. Quality scores used for our analysis reflect the total of majority score for each item on a given scale (Table 1).

The Downs and Jadad scales assess the quality of reports of randomized controlled trials. The modified Downs scale consisted of 27 questions with total scores ranging from 0–28. The Jadad scale consisted of three questions with a total score ranging from 0–5. Higher scores indicate higher report quality for both scales.

The full quality scores for each study are located in Appendix B and Appendix C of the supplemental information.

### Statistical analyses

Statistical analyses were performed using STATA version 11.0 (STATA Corp. College Station, Texas). Outcomes were analyzed on an intention to treat basis using random-effects models, as these models provide more conservative estimates than the fixed-effect model by incorporating both within- and between-study variations. We estimated the association between 25[OH]D and the primary outcome (BMI change) by calculating mean difference in BMI between baseline and study end with 95% confidence intervals calculated for each study. Summary measures of mean BMI differences were calculated using the *metan* program in STATA.

We tested the null hypothesis that all studies share a common effect size. Heterogeneity within studies was evaluated by the Cochran Q test, which has an X^2^ distribution with 7 degrees of freedom. A *p value* < 0.1 was considered statistically significant. To test for the true heterogeneity to total variance across the observed effect estimates, we also performed the I^2^ test. In addition, a random-effects meta-regression analysis was performed to test whether 25[OH]D supplementation was significantly associated with BMI change. All study characteristics that we examined were selected a priori. Selected characteristics were: 1) BMI higher than 30 or lower than 30; 2) average percent of females; 3) concomitant use of calcium; 4) follow-up duration; and 5) 25[OH]D dose.

Evidence of publication bias was investigated by means of funnel plot (Egger et al. and Begg and Mazumdar), in which the effect estimate (mean BMI difference) is plotted versus the standard error of the effect estimate. If there is no publication bias, the studies will be distributed symmetrically around the mean BMI difference (since the sampling error is random), with the more precise estimates tightly clustered around the mean and the less precise estimates more widely dispersed around the mean. The Begg and Mazamdar test is a rank correlation test of the null hypothesis that the studies’ estimates are not associated with their estimated errors. The test of Egger regressess the Z-score on the standard error. The expected intercept of this regression equation is zero when the estimates and their respective standard errors are not associated. Both tests evaluate funnel plot symmetry [12].

## Results

### Description of Studies Selected

Of the 30 studies reviewed [3,13–39], 9 were included in the meta-analysis (Table 2), including one article which was broken down by participant age group. In total, 1651 participants were analyzed, most of whom were women (n= 1401, [82.6%]) with a mean age of 47.9 years (SD ± 11.7). Mean follow-up varied between 6 to 192 weeks and mean daily dose of 25[OH]D supplementation varied from 200 IU to 1,110 IU per day, with most falling between 200 IU and 400 IU. Five of the 9 studies had calcium supplements in both the control and intervention groups [13–16] (between 800 mg to 1200 mg), while the other 4 studies did not use calcium supplements [17–19]. The mean BMI for the intervention and control groups were 30.7 kg/m^2^ (SD ± 3.2) and 30.4 kg/m^2^ (SD ± 3.3), respectively.

### Results of continuous data

**Figure 2:**
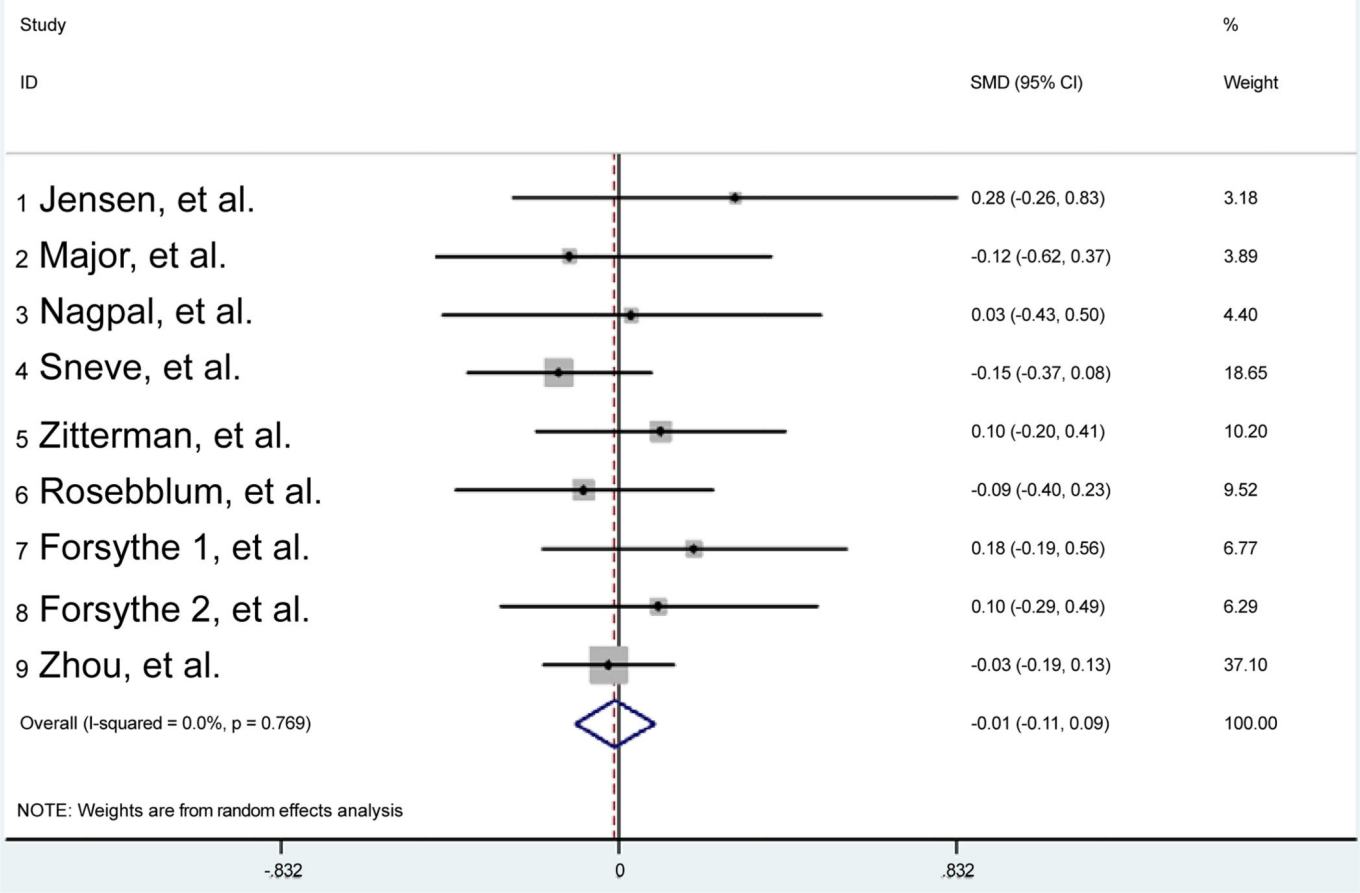
Forest plot of BMI change for each of the 9 trials weighted by 1/within-study variance using random effects model.

Meta-analysis showed no statistically significant differences between the treatment group (25[OH]D supplementation) and the control group (placebo) for BMI change. Figure 2 shows the forest plot of the random effects model, displaying BMI change for each of the 9 controlled trials weighted by 1/within- study variance. According to the protocol we proceeded to compare the ending values of the two groups (mean differences in BMI). The point estimates and the 95% confidence intervals were similar in the majority of the studies with one value skewed to the left widely. Heterogeneity was significant *p value* (p<0.0001). Of the 9 studies, five suggested a beneficial effect of 25[OH]D supplementation for the end point of BMI change (weight loss), whereas 3 showed a negative effect (weight gain) and one with non-perceptible change.

**Figure 3:**
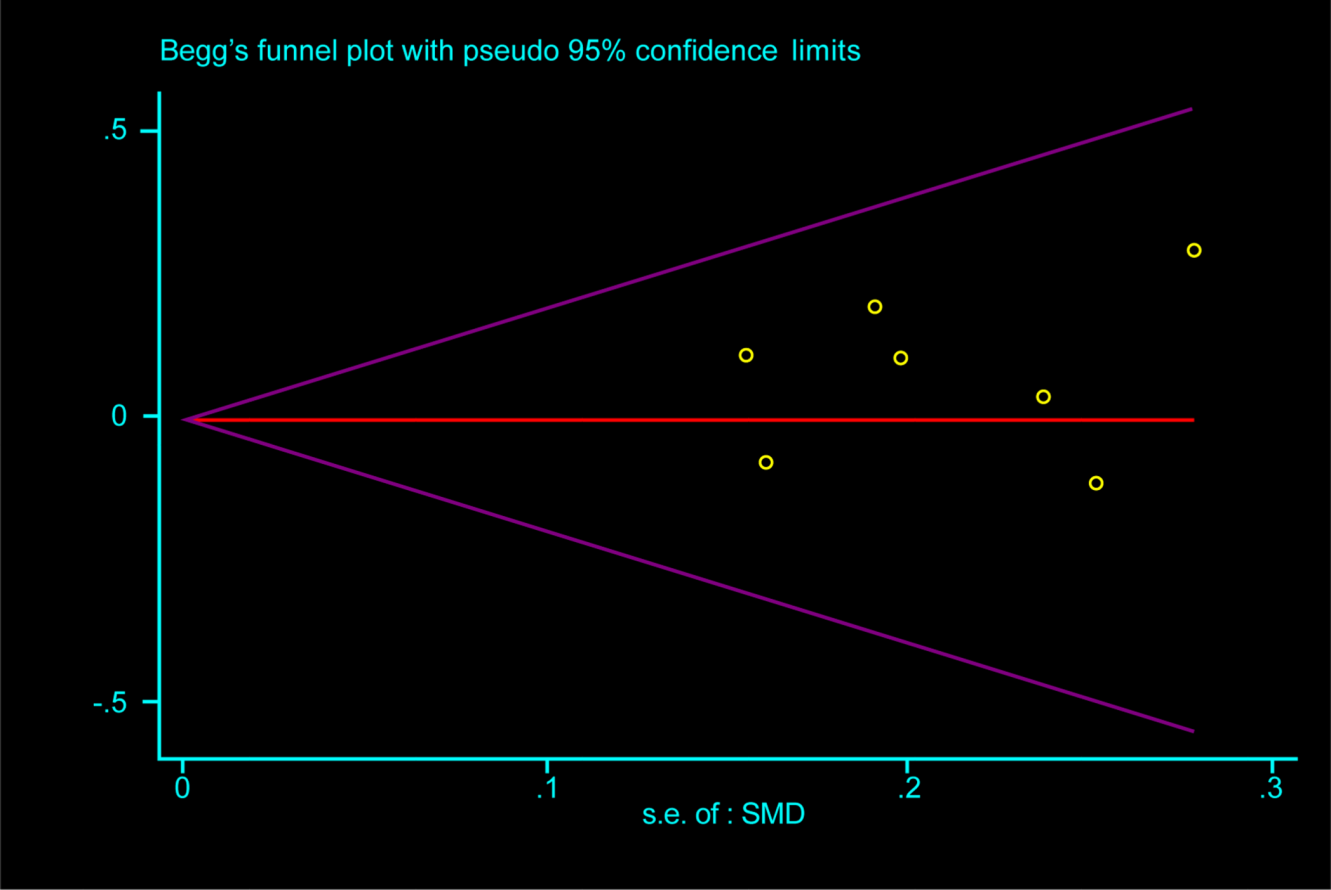
Funnel plot with pseudo 95% confidence limits for each of the 9 trials to assess publication bias in the association between change in BMI and vitamin D supplementation.

**Figure 4:**
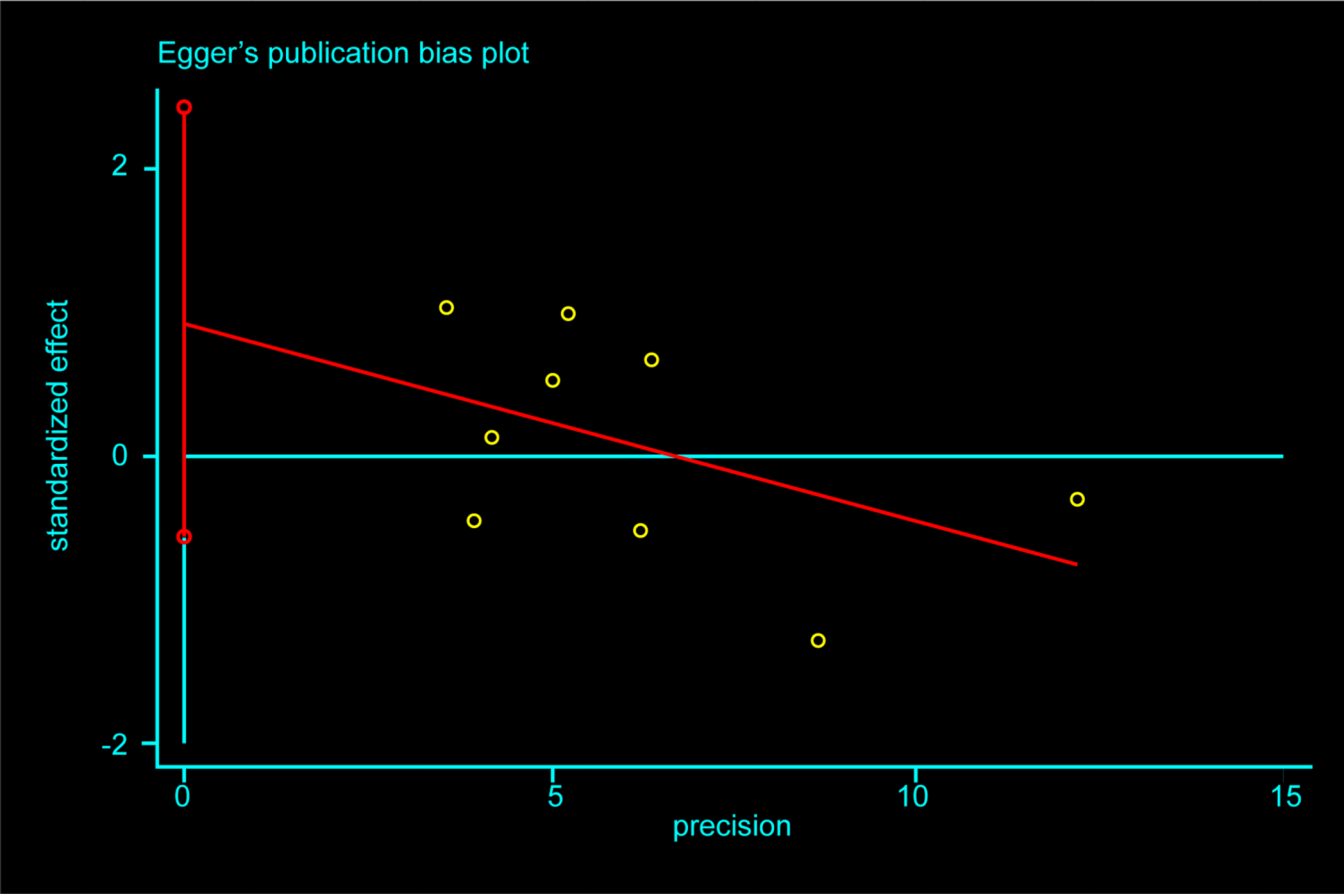
Egger plot to assess publication bias in the association between change in BMI and vitamin D supplementation.

Meta-analysis showed no statistically significant differences in BMI change by use of 25[OH]D supplementation (*p value*=0.8). The I^2^ showed that 0% of the effect of variance in dispersion in the true effects, and the T^2^ reflected no heterogeneity between studies on the absolute scale. Figure 3 displays the funnel plots which suggested no evidence of publication bias. The test of Begg and Mazumdar, and of Egger suggested no publication bias, both with a p value of 0.18 (Figure 4).

Two univariate linear meta-regressions were performed, using the 9 trials. As expected, no significant association was noted between 25[OH]D supplementation and BMI change after controlling for BMI or percentage of female participants in the study. Therefore, additional analyses sub-divided the studies according to baseline BMI values (i.e. high and low groups). Although not statistically significant, there may have been a trend towards a beneficial effect of 25[OH]D supplementation in subjects with higher baseline BMI values. Similar results were observed using a meta-regression analysis. No association was noted between 25[OH]D supplementation and BMI change after adjusting for baseline BMI groups (Adjusted R^2^=0%, I^2^=0%). Adjusting for baseline BMI as a continuous variable also demonstrated no associations between 25[OH]D supplementation and BMI change (R^2^=0%, I^2^=0%) (Table 3).

Each analysis adjusted for one covariate only due to limited number of studies (+BMI as a continuous variable, *prevalence of BMI ≥ 30 kg/m^2^ and percentage of female study participants). Beta-coefficient represents the adjusted summary estimate of BMI change by use of vitamin D supplementation vs. no vitamin d supplementation. A negative beta-coefficient indicates a decrease in BMI with use of vitamin D supplementation after adjusting for the covariate. All 95% confidence intervals (CI) include zero indicating no significant difference in BMI change by use of vitamin D supplementation after adjusting for the covariate.

A sensitivity analysis was performed including only high quality studies based on quality assessment scores (Downs scores ≥ 23 and Jadad scores ≥ 4 were included). Again, the results showed no statistically significant differences between the intervention and control groups for BMI change.

## Discussion

Based on the published literature to date, our pooled results indicate that oral 25[OH]D supplementation does not impact BMI change. Neither fixed-effect nor random effects meta-analyses showed any significant associations between 25[OH]D supplementation and BMI change. Specifically, in the fixed-effect analysis, we observed no variance in the differences between true values (I^2^=0, p=0.7 for heterogeneity chisquared test), suggesting that the true effect was the same in all of the studies. Findings were similar with the use of a random-effects analysis. Further analyses which adjusted for the percentage of females in the study, the dose of 25[OH]D supplementation, the co-administration of calcium, or the length of follow-up revealed no contribution of these factors to the potential association between 25[OH]D and BMI change. Most studies included populations with at least 50% females, and only one study focused on men. Gender does impact one’s ability to lose weight [40–43] and perhaps heterogeneity of effects exists between different genders. In contrast, the 25[OH]D dose was fairly consistent across studies, except for one outlier [17] The appropriate dose of 25[OH]D supplementation necessary to significantly impact BMI change remains unknown, and it is possible that higher doses of 25[OH]D could be more effective. Calcium has been suggested as another potential weight-loss aid [3,7,8] and the addition of this supplement in some of the studies at various doses further confounds the potential effects of 25[OH]D supplementation. Although calcium and 25[OH]D have been shown to increase whole body fat oxidation after meals [3] the exact effect of calcium on body weight, however, remains controversial [3,22,24–26].

One factor not measured in most studies was the baseline 25[OH]D level of study participants. Evidence suggests that a substantial number of adult Americans have low levels of 25[OH]D [44], and low serum levels of 25[OH]D have been shown to be inversely correlated with higher BMI values [21,45,46] and abnormal cardiovascular risk markers [47,48]. A higher baseline 25[OH]D level has also been correlated with an enhanced response to hypocaloric diets and loss of body fat in women [21]. Evidence from the 4th Tromso study in Norway, which included nearly 20 000 participants, suggests that dietary 25[OH]D intake may inversely correlate with BMI [46]. Shahar et al. [22] demonstrated that increases in serum 25[OH]D levels were associated with an increase in the likelihood of diet-induced weight loss [22]. Furthermore, an adequate response of 25[OH]D levels to oral supplementation may be inversely associated with baseline BMI, suggesting that body size should be considered when determining the appropriate dose [23]. While 25[OH]D supplementation over a relatively short-period can normalize serum levels, the effects are not likely maintained upon cessation of supplementation [49,50]. Therefore, the benefits from 25[OH]D supplementation may require prolonged dosing in order to maintain the desired effect.

Other potential confounders which varied across studies included diet, physical activity, and instruction/counseling. Due to the limited number of studies identified, further sub-analyses were not feasible, although the contribution of these variables remains possible. Additional factors not specifically discussed in this subset of studies that may also play a role in BMI change include race, socioeconomic status, type of community (urban vs. rural), and education.

Our quality assessments of each study revealed significant variability in the specified criteria for each assessment. Nevertheless, adjusting for these factors using meta-regression analyses did not change our findings of no significant differences in BMI change by 25[OH]D supplementation. The small number of trials included also limited the number of variables that could be examined simultaneously in meta-regression. While our initial search yielded a larger number of studies, the trials that met our inclusion criteria markedly decreased with further scrutiny. Several studies appeared to evaluate 25[OH] D supplementation and BMI change, but the data were presented as change in weight, adiposity, waist circumference or specific follow-up data needed for analysis were not provided [23,27–29]. Most studies reviewed focused on BMI and not on weight or other adiposity measures. BMI reflects both fat mass and muscle mass and may not be the best measure of adiposity. One randomized, double-blinded, placebo-controlled trial of >36,000 women demonstrated a small effect of calcium plus cholecalciferol on weight gain over several years [28]. The exclusion of quality studies such as this may have influenced our findings. Additionally, our findings may not be generalizable to non-obese populations since most studies selected for analyses included only obese populations.

## Conclusions

This study represents the first meta-analysis to evaluate the association between 25[OH]D supplementation and BMI change. Our extensive evaluation of each study, including two independent quality assessments, yielded a thorough and important review of the current literature. Although no significant association between oral 25[OH]D supplementation and BMI change was noted, the possibility of a small (undetected) correlation remains possible. Larger studies would be required to fully explore a potential smaller association, which could potentially add to our limited understanding of the complexities of weight management.

## Figures and Tables

**Table 1:** Quality assessment scores for selected articles

Study		
Jensen et al. [13]	14	2
Major et al. [14]	23	5
Nagpal et al. [17]	25	4
Sneve et al. [15]	21	4
Zitterman et al. [18]	24	5
Rosenblum et al. [16]	21	5
Forsythe et al. [19]	23	2
Zhou et al. [20]	24	5

Downs and Black [11]

**Table 2:** Summary of articles selected for analysis.

Study ID	Source	Year	Average Age	Ethnicity	Duration (weeks)	Total Participants	Treatment	Dwelling	Treatment Group BMI (SD)	Control Group BMI (SD)
1	Jensen et al. [13]	2001	Not reported	NE	12	52 (All Women)	200 IU of Cholecalciferol and 800 mg Calcium per day for 12 weeks	Outpatient Clinic	Baseline BMI of 34.8(1.1) and 2(1) unit decrease in BMI after 12 weeks	Baseline BMI of 34(1) and 2.3(1.1) decrease in BMI over 12 weeks
2	Major et al. [14]	2007	43.6	NE Quebec population	15	63 (All Women)	400 IU of Cholecalciferol and 1200 mg of Calcium per day for 15 weeks	Community	Baseline BMI 31.4(2.5) and 1.6(2.8) unit decrease in BMI after 15 weeks	Baseline BMI of 32.3(3.54) and 1.2(3.7) decrease in BMI over 15 weeks
3	Nagpal et al. [17]	2008	42.4	NE Indian	6	71 (All Male)	120000 IU of Cholecalciferol 3 doses every 2 weeks (8571 IU/day)	Community	Baseline BMI 26.7(4.54) and 0.02(.62) unit decrease in BMI after 6 weeks	Baseline BMI of 26(3.46) and 0.04(.68) decrease in BMI over 6 weeks
4	Sneve et al. [15]	2008	46.4	NE Norway	52	334 (62.7 % female and 37.3% male)	Either 20,000 or 40,000 IU of Cholecalciferol per week and 500 mg calcium for 12 months	Community and Outpatient Clinic	Baseline BMI for 40000 IU group 35.0(4.1) and 0(1.3) unit decrease in BMI and 20000 IU group 34.4(3.9) and 0.1(1.1) unit increase in BMI over 1 year	Baseline BMI of 35.1(3.8) and 0.2(1.4) increase in BMI over 1 year
5	Zitterman et al. [18]	2009	47.4	NE Germany	52	165 (62.2% Female and 37.8% male)	3332 IU of Cholecalciferol per day for 1 year	Community	Baseline BMI 33.7(4.1) and 2(2) unit decrease in BMI after 1 year	Baseline BMI of 33(4.3) and 2.2(1.9) decrease in BMI over 1 year
6	Rosenmblum et al. [69]	2012	39	White 75% Black 10% Hispanic 5.5% Other 9%	16	154 (91% Female and 9% men)	Orange Juice 3 times a day w/ 100 IU Cholecalciferol and 350 mg Calcium for 16 weeks	Community	Baseline BMI 30.4(2.7) and 1.1(1.3) unit decrease in BMI after 16 weeks	Baseline BMI of 29.9(2.7) and 1(1) decrease in BMI over 16 weeks
7	Forsythe et al. [19]	2012	28	NE Ireland	22	110 (Both Female and male) age 20–40	15ug of Cholecalciferol per day for 22 weeks	Community	Baseline BMI 26.23(4.48) and .23(.07) unit increase in BMI after 22 weeks	Baseline BMI of 25.89(4.24) and .11(.6) increase in BMI over 22 weeks
8	Forsythe et al. [19]	2012	71	NE Ireland	22	102 (both female and male) 65 and older	15ug of Cholecalciferol for 22 weeks	Community	Baseline BMI of 29.24(4.36) and .23(.58) unit increase in BMI after 22 weeks	Baseline BMI of 28.72(4.25) and .17(.64) increase in BMI over 22 weeks
9	Zhou et al. [20]	2010	65.8	White Non-hispanic USA	192	632 (100% female)	1100 IU Cholecalciferol and 1400mg Calcium per day for 4 years	Community	Baseline BMI 28.8(5.49) and 0.05 (0.48) unit decrease after 192 weeks	Baseline BMI 28.8(5.48) and 0.06(0.1) unit increase after 192 weeks

**Table 3:** Results of univariate meta-regression on summary estimates of BMI change by use of vitamin D supplementation

Covariate	Βeta-coefficient	Standard error	95% CI	R^2^
+BMI	−0.010	0.010	−0.055 0.026	0
*BMI ≥ 30	−0.060	0.090	−0.300 0.170	0
Female % average	−0.001	0.002	−0.006 0.003	0
